# Comparison of the Size Measurement of Gallbladder Polyps by Three Different Radiologists in Abdominal Ultrasonography

**DOI:** 10.3390/tomography10070077

**Published:** 2024-07-03

**Authors:** Kyu-Chong Lee, Jin-Kyem Kim, Dong-Kyu Kim

**Affiliations:** 1 Department of Radiology, Armed Forces Capital Hospital, Seongnam 13574, Republic of Korea; mikarune@naver.com (K.-C.L.); kyem88@naver.com (J.-K.K.); 2 Department of Radiology, Korea University Anam Hospital, 73 Geryeodae-ro, Seongbuk-Gu, Seoul 02841, Republic of Korea; 3 Department of Radiology, Severance Hospital, Research Institute of Radiological Science, Yonsei University College of Medicine, 50-1 Yonsei-Ro, Seodaemun-Gu, Seoul 03722, Republic of Korea

**Keywords:** gallbladder, polyps, ultrasonography, reproducibility of results

## Abstract

Background: There is little information regarding the size measurement differences in gallbladder (GB) polyps performed by different radiologists on abdominal ultrasonography (US). Aim: To reveal the differences in GB polyp size measurements performed by different radiologists on abdominal US. Methods: From June to September 2022, the maximum diameter of 228 GB polyps was measured twice on abdominal US by one of three radiologists (a third-year radiology resident [reader A], a radiologist with 7 years of experience in abdominal US [reader B], and an abdominal radiologist with 8 years of experience in abdominal US [reader C]). Intra-reader agreements for polyp size measurements were assessed by intraclass correlation coefficient (ICC). A Bland–Altman plot was used to visualize the differences between the first and second size measurements in each reader. Results: Reader A, reader B, and reader C evaluated 65, 77, and 86 polyps, respectively. The mean size of measured 228 GB polyps was 5.0 ± 1.9 mm. Except for the case where reader A showed moderate intra-reader agreement (0.726) for polyps with size ≤ 5 mm, all readers showed an overall high intra-reader reliability (reader A, ICC = 0.859; reader B, ICC = 0.947, reader C, ICC = 0.948), indicative of good and excellent intra-reader agreements. The 95% limit of agreement of reader A, B, and C was 1.9 mm of the mean in all three readers. Conclusions: GB polyp size measurement on abdominal US showed good or excellent intra-reader agreements. However, size changes of approximately less than 1.9 mm should be interpreted carefully because these may be within the measurement error.

## 1. Introduction

Gallbladder (GB) cancer is the most common malignancy of the biliary tract and is associated with poor prognosis with a 5-year survival rate of 5% [1,2,3]. Since some GB cancers can arise from pre-existing GB polyps, patients with GB polyps undergo periodical follow-up ultrasonography (US) to observe changes in polyp size or shape and to offer early curative treatment by early diagnosis of GB cancers. In the adult population, the estimated prevalence of GB polyps ranges from 0.3% to 9.5%, and GB polyps are often detected incidentally on abdominal US examinations [4,5].

Abdominal US has been widely accepted as a primary modality for GB polyp size measurement and follow-up. While the majority of GB polyps are benign, a small portion of them are genuine neoplastic polyps, which carry a small but unknown risk of turning malignant. Distinguishing between nonneoplastic and neoplastic polyps through imaging poses a challenge. Additionally, only 6% of GB cancers may stem from a specific type of precursor lesion, with the majority of GB cancers developing from a flat dysplastic epithelium. Even in GB polyps larger than 10 mm, some studies indicate that only 0.4% are malignant, and the majority of malignant polyps are typically larger than 20 mm [6,7,8,9,10,11]. As more evidence emerges, there is a growing tendency to question existing management guidelines given that the vast majority of surgically removed GB polyps are found to be non-cancerous [12,13]. Taking an aggressive approach in handling small GB polyps can potentially harm patients, resulting in unnecessary surgical removal, and frequent and extended follow-up imaging with uncertain benefits, as well as causing anxiety and inconvenience for the patients [14].

A recent extensive population study discovered that the rate of GB cancer in patients with GB polyps identified through US was the same (0.053% [19 out of 35,856 patients]) as in those without GB polyps (0.054% [316 out of 586,357 patients]). Moreover, individuals with GB cancer exhibited a similar occurrence of concurrent GB polyps (6.0% [22 out of 365 patients]) compared to those without GB cancer (5.8% [35,856 out of 622,227 patients]). Consequently, there seems to be no elevated relative risk of developing GB cancer in individuals with asymptomatic GB polyps [13]. Furthermore, a recent investigation involving 156 patients with confirmed GB polyps histopathologically in four Dutch hospitals reported that GB polyp size was frequently overestimated on abdominal US examination. The study also concluded that the 10 mm threshold for surgical removal resulted in unnecessary treatment for nonneoplastic polyps [7]. In another recent research involving 434 patients with GB polyps detected on abdominal US examinations, the longitudinal changes in size of GB polyps on serial US examinations were evaluated. The results of this study showed that GB polyps fluctuated in size, number, and visibility over serial examinations and no GB cancer was identified during the study period, though the increase in size by 2 mm or more was frequent [15]. Therefore, the study suggested that the recommended threshold of a 2 mm growth, as proposed by the European multisociety guidelines [16], might be too conservative for justifying cholecystectomy. However, according to the recently updated guidelines for management of GB polyps, only a size change of 2–3 mm can make a difference in the management of polyps [14,17]. Therefore, reliable and accurate size measurement of GB polyps is important for proper management.

However, when performing abdominal US for GB polyps, there were many cases where the polyp size was slightly different even if the same radiologist measured the size of the same polyp in the same patient; thus, it was often challenging to distinguish between true growth and measurement error. However, there is little information regarding the size measurements differences in GB polyps performed by different radiologists on abdominal US [18]. Therefore, the aim of this study was to reveal the differences in GB polyp size measurements performed by different radiologists on abdominal US.

## 2. Materials and Methods

### 2.1. Study Population

This single-center retrospective study was approved by our institutional review board (institutional review board approval number: AFCH 2023-05-004). Given the retrospective nature of the investigation and the use of anonymized patient data, requirements for informed consent were waived. The US image data obtained between June 2022 and September 2022 were accessed for research purposes from 1 March 2023 to 31 May 2023.

**Figure 1:**
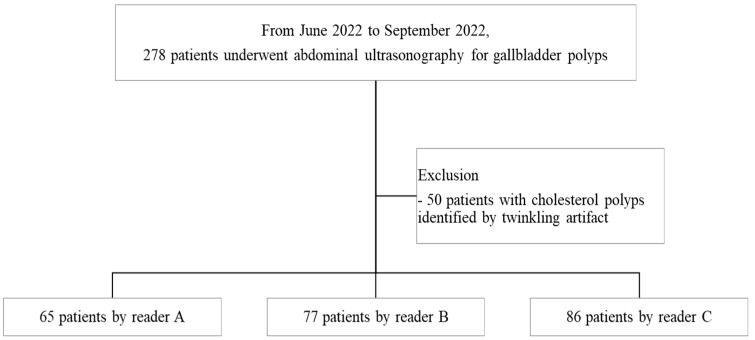
Flow diagram of study patients in this study.

From June 2022 to September 2022, there were 278 patients who underwent abdominal US examination for GB polyps in our hospital. Among them, fifty patients with cholesterol GB polyps that were difficult to differentiate from other GB pathologies such as combined GB stones/sludge, as identified by the twinkling artifact, were excluded. Finally, a total of 228 patients who underwent abdominal US performed by one of three radiologists (a third-year radiology resident [reader A], a radiologist with 7 years of experience in abdominal US [reader B], and an abdominal radiologist with 8 years of experience in abdominal US [reader C]) were included in this study (Figure 1).

### 2.2. Image Acquisition of GB Polyps

US was performed in all 228 patients with a 2–5 MHz convex transducer (iU22, Philips Medical Systems, Bothell, WA, USA). All US images were obtained by using the preloaded artifact/noise suppression software XRES and sonoCT with harmonic imaging techniques. The patients fasted for a minimum of 8 h before undergoing abdominal US. The US examinations were conducted with patients in the supine position, and if necessary, in the left lateral decubitus position, taking into account body habitus and bowel gas. The polyp was determined as the solid, hyperechoic, non-shadowing, and non-mobile lesion arising from the GB wall. The largest size of polyp (largest diameter at any plane) was measured using an electronic ruler. For each session, the size of polyps was measured twice on a window setting by each reader. To reduce recall bias, each reader scanned the GB first and then the other organs, including the liver, bile duct, pancreas, and spleen, after which the GB was scanned again. Based on the time when the first GB polyp image and the last GB polyp image was obtained, the interval periods (minutes) between the first and second US evaluations of GB polyps were calculated.

In our institution, there was an established internal regulation stating that it was acceptable to measure GB size only once during the US examination. However, the two radiologists involved in this study were individuals who routinely measure GB size twice during US examinations and the radiologist resident who received training from these individuals also conducted US examinations in a similar manner. All measurements aimed at measuring the maximal diameter, and all other conditions were kept consistent.

### 2.3. Data Acquisition of Study Patients

**Figure 2:**
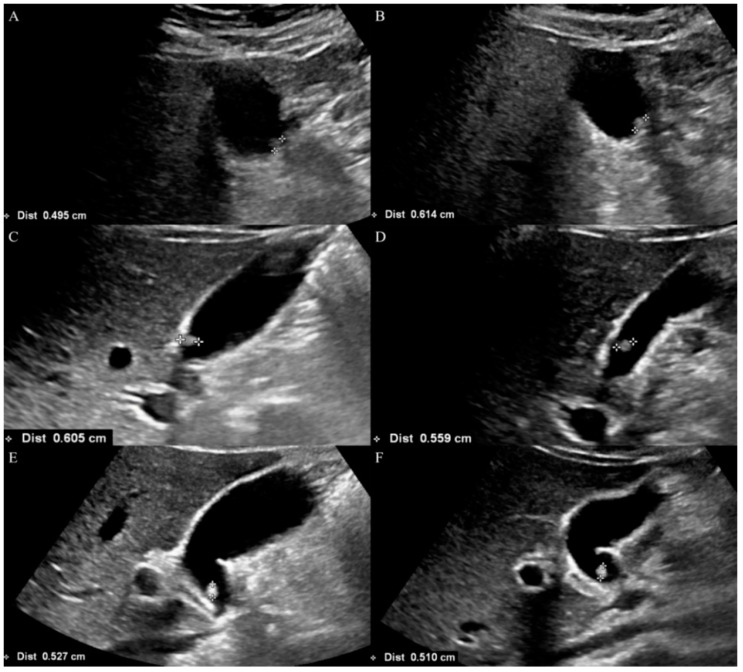
Representative cases of gallbladder polyp size measurements in each reader. (**A**,**B**) A polyp measured twice by reader A. The mean size of the polyp was 5.5 mm. The size difference (mm) between the measurements was 1.2 mm. (**C**,**D**) A polyp measured twice by reader B. The mean size of the polyp was 5.8 mm. The size difference (mm) between the measurements was 0.5 mm. (**E**,**F**) A polyp measured twice by reader B. The mean size of the polyp was 5.2 mm. The size difference (mm) between the measurements was—0.2 mm.

Clinical data including patients’ age and sex at the time of US examinations were obtained from electronic medical charts. The location, type, and size of GB polyps were recorded by retrospectively reviewing the US images on the ZeTTA PACS Viewer 2001 (Taeyoung Soft, Anyang, Republic of Korea). If there were multiple GB polyps, only the largest lesion was registered. The mean size of the GB polyp in each patient was calculated as follows: (first size measurement + second size measurement)/2 (Figure 2). Then, the size of polyps was divided into two groups: mean size ≤ 5 mm vs. >5 mm. The location of polyps was classified as neck, body, and fundus. The type of polyps was classified as sessile, pedunculated, and too small to characterize. The terms were defined as follows: pedunculated = point of attachment to the wall is via a stalk or pedicle; sessile = flat or dome-shaped mass that extends out from the mucosal layer and does not have a stalk; point of attachment to wall is broad-based. The pathologic reports of the patients who underwent cholecystectomy were also reviewed. The decision of whether to perform a cholecystectomy was left to the discretion of the surgeon.

### 2.4. Statistical Analysis

Continuous variables were expressed as mean ± standard deviation (SD) and categorical values were expressed as absolute numbers with percentages. Intra-reader agreements for continuous variables were assessed by intraclass correlation coefficient (ICC). ICC results were interpreted as follows: poor (ICC < 0.50), moderate (0.50–0.75), good (0.75–0.90), and excellent (ICC > 0.90) [19]. A Bland–Altman plot was used to visualize the differences between the first and second polyp size measurements in each reader. Statistical analyses were performed using IBM SPSS Statistics for Windows, version 27.0 (IBM Corp., Armonk, NY, USA). A *p* value of less than 0.05 was considered statistically significant.

## 3. Results

### 3.1. Baseline Characteristics of GB Polyps

Of the 228 GB polyps in 228 patients (mean age = 43.8 ± 7.8 years, 189 men and 39 women), reader A, reader B, and reader C evaluated 65, 77, and 86 polyps, respectively. The mean size of the 228 GB polyps was 5.0 ± 1.9 mm. Among them, 138 polyps (60.5%) were ≤5 mm in size, while the remaining 90 polyps (39.5%) were larger than 5 mm. The polyps were located as follows: in the neck (*n* = 92, 40.4%), body (*n* = 121, 53.1%), and fundus (*n* = 15, 6.6%) of the GB. Regarding type, they were categorized as sessile (*n* = 30, 13.2%), pedunculated (*n* = 160, 70.2%), and too small to characterize (*n* = 38, 16.7%). The mean interval periods between the first and second US evaluation in all three readers were 8.4 ± 1.5 min. Further details on the baseline characteristics of the GB polyps are summarized in Table 1.

### 3.2. Intra-Reader Agreements for Size Measurements of GB Polyps

Among the study patients, there were 12 patients who underwent cholecystectomy. The reported pathologic results of these patients were chronic cholecystitis with cholesterolosis. The reported maximal diameters of GB polyps on pathologic tissue are summarized in Table 2.

Table 3 shows the intra-reader agreements of the GB polyp size measurements in each reader. The ICCs for reader A, reader B, and reader C were 0.859 (95% confidence interval [CI], 0.774–0.911), 0.947 (95% CI, 0.917–0.966), and 0.948 (9 5% CI, 0.920–0.966), respectively. Reader A showed overall lower intra-reader agreements (good, 0.859) for size measurements compared with reader B and C (excellent, 0.947 and 0.948). Furthermore, according to the 5 mm cutoff of size, reader A showed moderate intra-reader agreement (0.726) for polyps with size ≤ 5 mm, while the other readers showed good intra-reader agreements (0.808 and 0.840). With the exception of this, all readers showed good or excellent intra-reader agreements for polyp size measurements regardless of location and type.

**Figure 3:**
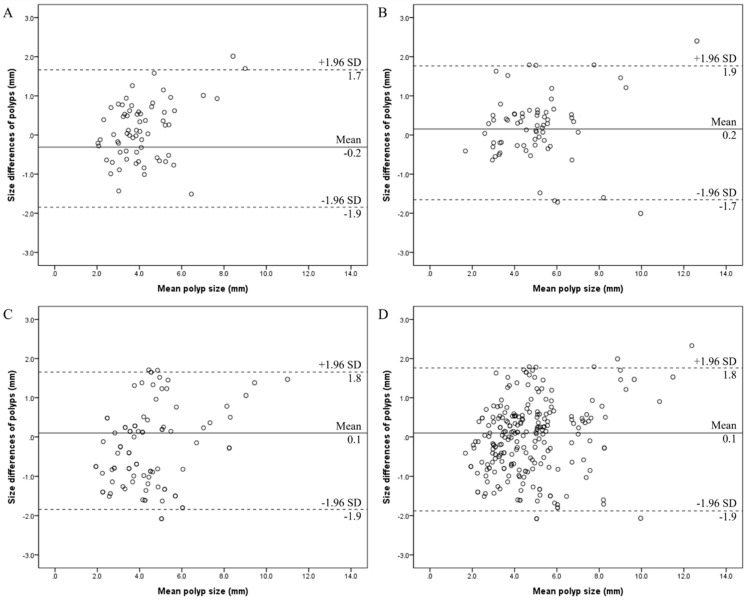
Bland–Altman plots show the intra-reader agreements of the gallbladder polyp size measurements for reader A (**A**), reader B (**B**), and reader C (**C**). A Bland–Altman plot of all 228 gallbladder polyps is also depicted (**D**). X-axis = the mean of the measurements of polyp size, Y-axis = size differences between the measurements (mm of the mean), solid line = mean size difference (mm), and dashed line = 95% limit of agreement.

Figure 3 depicts Bland–Altman plots of polyp size measurements in each reader. The 95% limit of agreement of each reader was 1.9 mm of the mean in all readers. Moreover, the 95% limit of agreement of all 228 GB polyps was also 1.9 mm.

## 4. Discussion

The results of the present study underscore the complexities involved in the evaluation and measurement of GB polyps while also highlighting potential areas for further investigation and improvement in clinical practice by evaluating intra-reader reliability for GB polyp size measurements on abdominal US. The demographic characteristics of the study population, including the mean age and gender distribution, reflect the typical demographics of patients presenting with GB polyps. The predominance of males in the study population is consistent with previous reports suggesting a higher prevalence of GB polyps in men. The distribution of polyps across different size categories highlights the prevalence of smaller polyps, with approximately 60.5% of polyps being ≤5 mm in size. This is consistent with the existing literature, indicating that the majority of GB polyps are small and have a benign course. However, it is important to note that a considerable proportion of polyps were larger than 5 mm, underscoring the significance of accurate sizing in distinguishing between benign and potentially malignant lesions. The anatomical distribution of polyps within the GB provides additional context for their evaluation and management. The majority of polyps were located in the body and neck of the GB, with a smaller proportion found in the fundus. This distribution may have implications for the approach to imaging and surveillance protocols, as well as the risk stratification of polyps based on their location within the GB. The classification of polyps by type further elucidates the heterogeneity of these lesions, with the majority being pedunculated. Sessile polyps were less common, while a significant proportion were too small to characterize. This highlights the challenges associated with accurately characterizing smaller polyps, which may necessitate additional imaging modalities or surveillance strategies for further evaluation.

In this study, all readers showed overall high intra-reader reliability (ICCs between 0.859 and 0.948), indicative of good and excellent intra-reader agreements. However, reader B and C showed overall higher intra-reader agreements (excellent, ICC = 0.947 and 0.948) than reader A (good, ICC = 0.859). The 95% limit of agreement within the same reader ranged from 1.7 mm to 1.9 mm. In a previous study [18], the inter-reader and intra-reader reliability for measurements of GB polyps were assessed. However, the previous study is based on the analysis of 91 polyps by two readers; hence, the sample size was small. Additionally, to our best knowledge, previous studies on intra-reader reliability for GB polyp size are scarce, making this study unique and serving as further validation. Furthermore, the present study calculated the “mm” discrepancies instead of “%” discrepancies. If the differences in GB polyp size measurements were expressed as “%”, even with the same percentage difference, the actual “mm” differences would increase as the size of the GB polyp increases because the percentage discrepancy depends on size; for instance, a 1 mm difference would represent a 20% discrepancy for a 5 mm polyp but only a 10% difference for a 10 mm polyp. Therefore, “mm” discrepancies were calculated instead of % discrepancies in this study and the results showed a 95% limit of agreement of 1.9 mm, which differs from the previous study. This strongly indicates that GB polyp size changes of less than 2 mm should be interpreted with great caution, as the 95% limit of agreement was 1.9 mm for all operators, regardless of whether the specialized radiologist or less experienced radiology resident performed the US examination. Lastly, there are not many studies that directly compared the size of GB polyps in the pathologic report after cholecystectomy with the size of GB polyps on pre-operative US examination for each patient. In our study, although the number of patients who underwent cholecystectomy was small, with only 12 individuals, the direct comparison between the pathologic tissue and the US findings was performed in each patient and the results showed that the difference in GB polyp size between radiology and pathology was also not greater than 2 mm.

Recently, the guidelines for management and follow-up of GB polyps were updated, and the management of GB polyps differed according to the risk factors of malignancy, type of polyps (sessile or pedunculated), combined focal wall thickening, etc. However, generally, if the patients had no risk factors for malignancy, follow-up is not required for a GB polyp of 6 mm or less; periodical US follow-up is recommended for a polyp with size of 6–9 mm, and cholecystectomy is recommended for a polyp with size more than 10–15 mm. Furthermore, in the case a polyp grows by more than 2 mm within the 2-year follow-up period, the patient’s risk factors should be considered with the current polyp size [14,17]. Furthermore, according to the guidelines for GB polyps based on the European Society of Gastrointestinal and Abdominal Radiology (ESGAR), European Association for Endoscopic Surgery and other Interventional Techniques (EAES), International Society of Digestive Surgery–European Federation (EFISDS) and European Society of Gastrointestinal Endoscopy [17], accurate and reliable size measurements of GB polyps are important since a size difference of only about 1–2 mm can change management. The result of our study, showing 95% limit of agreement of 1.9 mm, are in line with the guidelines, suggesting taking into account the current size of the lesion along with patient risk factors in cases where the polypoid lesion grows by 2 mm or more within the 2-year follow up period.

Transabdominal US is commonly employed as the primary tool for measuring and monitoring GB polyps. High-resolution US or contrast-enhanced US proves beneficial for assessing the risk associated with GB polyps [20,21]. However, there is always some inherent measurement error. Furthermore, a recent systematic review indicated that 93% of polyps did not exhibit an increase in size over long-term follow-up, suggesting that not all observed size changes during the follow-up period may be accurate reflections of actual size changes. Nonetheless, while rapid size increases are rare, they seem to pose a potential risk factor for malignancy [22,23]. Therefore, it is crucial to differentiate between measurement error and genuine size changes during the follow-up process. The 95% limit of agreement delineates the range in which 95% of all size changes attributed to measurement errors occur. If the size change falls within the 95% limit of agreement, it cannot be confidently regarded as a genuine change with a 95% CI [24]. In our study, the 95% limit of agreement within the same observer was approximately 1.9 mm. As repeated measurements over a brief time period with the same imaging operator, imaging unit, and settings were conducted in this study, the variability in size is likely indicative of measurement error. Therefore, a polyp size change of less than 1.9 mm might be within the measurement error and would not be considered a true change in size. According to the consensus guidelines for GB polyps, follow-up US is advised for polyps measuring 9 mm or less and without symptoms or risk factors [14]. If polyps show an increase of 2 mm or more during follow-up, cholecystectomy is recommended. The results of this study may offer supporting evidence for these guidelines, particularly regarding changes in polyp size over time.

The categorization of polyps based on size, location, and type further elucidates the complexity of GB polyp assessment. The majority of polyps in this study were smaller than or equal to 5 mm, with a significant proportion being pedunculated. However, it is worth noting that a considerable number of polyps were too small to characterize, indicating the limitations of current imaging modalities in accurately characterizing these lesions. The distribution of polyps across different regions of the GB also highlights the importance of thorough examination and consideration of anatomical variations in the evaluation process. The size of GB polyps can affect size measurement accuracy, as demonstrated by the results of this study. In the present study, intra-reader agreement in measuring polyp with a size > 5 mm was superior to that of ≤5 mm in each reader. Therefore, care should be taken when measuring small polyps in clinical practice. It was also evaluated that if there were other factors affecting measurement reliability. However, all readers showed good or excellent intra-reader agreements for polyp size measurements regardless of location and type. Furthermore, the Bland–Altman plots presented in Figure 3 provide additional insights into the agreement and discrepancies between readers in polyp size measurements. The narrow 95% limit of agreement observed across all readers indicates relatively consistent measurement discrepancies, with the majority falling within a clinically acceptable range of ±1.9 mm. This suggests that while there may be variability in absolute size measurements between readers, it is unlikely to significantly impact clinical decision-making regarding the management of GB polyps. Since the measurements were repeated on the same day with the same imaging reader, imaging settings, and equipment, the variability in polyp size may reflect the measurement error. Therefore, size changes of approximately less than 1.9 mm should be interpreted carefully because these may be measurement errors and may not be true size changes.

Though all readers showed overall high intra-reader reliability (good or excellent) for polyp size measurements on abdominal US, reader A, a third-year training resident, showed lower intra-reader agreement (ICC = 0.859) than readers B (ICC = 947) and C (ICC = 0.948), who are radiology specialists. It is a well-known limitation of US examination that the result of US is dependent on the examiner and examiner’s experience [25,26]. This suggests that there may be variability in the measurement techniques or interpretations employed by different readers, highlighting the importance of standardization and training in polyp assessment. Moreover, the moderate intra-reader agreement observed for polyps ≤ 5 mm in size by reader A underscores the challenges associated with accurately sizing smaller polyps, which are known to pose diagnostic dilemmas due to their increased likelihood of being benign. Therefore, to perform accurate and reliable US examinations, appropriate training by the staff with more clinical experience is required for training residents. In addition, there was moderate intra-reader agreement only when reader A measured polyps with a size ≤ 5 mm; thus, more careful interpretation is needed when the size changed in small polyps.

There were several limitations in this study. The first was its retrospective design and inherent selection bias; moreover, there were only 12 patients with pathologic results for GB polyps resected by cholecystectomy. Therefore, a limitation of this study was that only 12 out of the 228 patients could be used as a control group for comparing the GB polyp size measured in US examinations; so, statistical analysis of GB polyp size measured by three different radiologists with a control group cannot be performed. However, despite the small number of patients, the pathology data obtained from these 12 patients were added and compared with the radiology data obtained from US examination, and the results of this study showed that the difference in GB polyp size between real tissue and imaging recording was also of less than 2 mm. Comparative studies with a larger number of patients with pathology data as a control group are needed to strengthen the validity of our research findings. Second, the number of study patients assigned to each reader was relatively small. To validate the reliability of polyp size measurement, a further study with a large sample size is needed. Third, the inter-reader agreement was not assessed since the readers did not each perform the US examination for the same patient group; so, overall inter-reader and intra-reader reliability for all 228 GB polyps cannot be analyzed. Therefore, further prospective studies, with patient consent obtained prior to implementation, are necessary. Fourth, the size of polyps was measured twice within a short interval by each reader. Even though the readers scanned the GB first and last after scanning the other organs to reduce recall bias, there may be some recall bias. However, in actual clinical practice, abdominal US was performed after the reader checked the patient’s previous images; thus, the short interval between the GB polyp size measurements may not weaken the clinical importance of our results. Fifth, this study was conducted in a single center with a specific patient population and using a particular US system and settings. Multi-center studies involving diverse populations and different imaging technologies may be needed to enhance the external validity of the results. In conclusion, this study contributes valuable information regarding the assessment and measurement of GB polyps, highlighting the need for standardized protocols and ongoing training to improve consistency among radiologists. Despite the observed inter-reader variability, the overall good-to-excellent intra-reader agreements suggest that current imaging techniques are reliable for polyp size assessment. Therefore, GB polyp size measurement on abdominal US is reliable with good or excellent intra-reader agreements. However, size changes of approximately less than 1.9 mm should be interpreted carefully because these may be within the measurement error. Further research is warranted to validate these findings in larger, more diverse patient cohorts and to explore potential strategies for improving the accuracy and reproducibility of GB polyp evaluation.

## Figures and Tables

**Table 1:** Baseline characteristics of the gallbladder polyps.

Pt. No	Age	Sex	Maximal Diameter on Pathology (mm)	Maximal Diameter on Ultrasonography (mm)	Reader	Size Difference between Radiology and Pathology Reports (mm)	Interval Periods (Days) *
1	44	Male	5.1	6.2	A	−1.1	92
2	42	Male	14.9	13.0	B	1.9	21
3	45	Female	9.1	8.2	A	0.9	66
4	41	Male	9.5	9.3	A	0.2	45
5	38	Male	13.0	11.5	C	1.5	28
6	40	Male	8.2	9.1	C	−0.9	28
7	52	Male	8.0	7.1	A	0.9	87
8	47	Male	11.3	10.0	B	1.3	25
9	39	Male	6.5	4.8	A	1.7	388
10	46	Male	10.3	9.6	B	0.7	54
11	48	Female	8.5	9.1	B	−0.6	48
12	51	Female	9.8	9.5	C	0.3	50

* Interval periods = periods (days) between the day of ultrasound examination and that of cholecystectomy.

**Table 2:** The pathologic reports of the patients who underwent cholecystectomy.

Characteristics	Reader A	Reader B	Reader C	Total
No. of patients	65	77	86	228
Age (years)	44.4 ± 8.0	41.6 ± 7.6	45.4 ± 7.4	43.8 ± 7.8
Sex, *n* (%)				
Men	54 (83.1)	67 (87.0)	68 (79.1)	189 (82.9)
Women	11 (16.9)	10 (13.0)	18 (20.9)	39 (17.1)
Size of polyps (mm)				
≤5 mm, *n* (%)	44 (67.7)	51 (66.2)	43 (50.0)	138 (60.5)
>5 mm, *n* (%)	21 (32.3)	26 (33.8)	43 (50.0)	90 (39.5)
Location of polyps, *n* (%)				
Neck	27 (41.5)	30 (39.0)	38 (44.2)	92 (40.4)
Body	35 (53.9)	44 (57.1)	39 (45.3)	121 (53.0)
Fundus	3 (4.6)	3 (3.9)	9 (10.5)	15 (6.6)
Type of polyps, *n* (%)				
Sessile	8 (12.3)	9 (11.7)	13 (15.1)	30 (13.2)
Pedunculated	47 (72.3)	58 (75.3)	55 (64.0)	160 (70.2)
N/A *	10 (15.4)	10 (13.0)	18 (20.9)	38 (16.6)

Continuous values are expressed as mean standard deviation. * N/A = not applicable (due to too small size to characterize).

**Table 3:** Intraclass correlation coefficients (ICCs) for gallbladder polyp size measurements in each reader according to the size, location, and type.

	Reader A	Reader B	Reader C
All measured polyps †	0.859 (0.774–0.911)	0.947 (0.917–0.966)	0.948 (0.920–0.966)
Size			
≤5 mm	0.726 (0.445–0.876)	0.808 (0.675–0.886)	0.840 (0.677–0.921)
>5 mm	0.821 (0.507–0.932)	0.920 (0.795–0.969)	0.902 (0.823–0.947)
Location			
Neck	0.924 (0.934–0.965)	0.933 (0.858–0.968)	0.936 (0.878–0.967)
Body	0.823 (0.605–0.925)	0.957 (0.921–0.976)	0.951 (0.910–0.973)
Fundus	0.934 (0.070–0.998)	0.901 (0.083–0.997)	0.964 (0.732–0.996)
Type			
Sessile	0.837 (0.536–0.938)	0.966 (0.758–0.995)	0.947 (0.673–0.992)
Pedunculated	0.875 (0.780–0.940)	0.920 (0.866–0.952)	0.941 (0.903–0.964)
N/A *	0.832 (0.470–0.938)	0.974 (0.903–0.994)	0.971 (0.905–0.992)

†—All ICCs are presented with 95% confidence interval in parentheses. * N/A = not applicable (due to too small size to characterize).

## Data Availability

The data that support the findings of this study are available from the corresponding author, [Dong-Kyu Kim], upon reasonable request.
